# BCL-3 expression promotes colorectal tumorigenesis through activation of AKT signalling

**DOI:** 10.1136/gutjnl-2014-308270

**Published:** 2015-06-01

**Authors:** Bettina C Urban, Tracey J Collard, Catherine J Eagle, Samantha L Southern, Alexander Greenhough, Maryam Hamdollah-Zadeh, Anil Ghosh, Richard Poulsom, Christos Paraskeva, Andrew Silver, Ann C Williams

**Affiliations:** 1 School of Cellular & Molecular Medicine, University of Bristol, Bristol, UK; 2 Centre for Digestive Diseases, National Centre for Bowel Research and Surgical Intervention, Blizard Institute of Cell and Molecular Science, Barts and The London School of Medicine and Dentistry, Queen Mary University of London, Whitechapel, London, UK

**Keywords:** NUCLEAR FACTOR KAPPA B, COLORECTAL CANCER, CANCER PREVENTION, APOPTOSIS, INFLAMMATION

## Abstract

**Objective:**

Colorectal cancer remains the fourth most common cause of cancer-related mortality worldwide. Here we investigate the role of nuclear factor-κB (NF-κB) co-factor B-cell CLL/lymphoma 3 (BCL-3) in promoting colorectal tumour cell survival.

**Design:**

Immunohistochemistry was carried out on 47 tumour samples and normal tissue from resection margins. The role of BCL-3/NF-κB complexes on cell growth was studied in vivo and in vitro using an siRNA approach and exogenous BCL-3 expression in colorectal adenoma and carcinoma cells. The question whether BCL-3 activated the AKT/protein kinase B (PKB) pathway in colorectal tumour cells was addressed by western blotting and confocal microscopy, and the ability of 5-aminosalicylic acid (5-ASA) to suppress BCL-3 expression was also investigated.

**Results:**

We report increased BCL-3 expression in human colorectal cancers and demonstrate that BCL-3 expression promotes tumour cell survival in vitro and tumour growth in mouse xenografts in vivo, dependent on interaction with NF-κB p50 or p52 homodimers. We show that BCL-3 promotes cell survival under conditions relevant to the tumour microenvironment, protecting both colorectal adenoma and carcinoma cells from apoptosis via activation of the AKT survival pathway: AKT activation is mediated via both PI3K and mammalian target of rapamycin (mTOR) pathways, leading to phosphorylation of downstream targets GSK-3β and FoxO1/3a. Treatment with 5-ASA suppressed BCL-3 expression in colorectal cancer cells.

**Conclusions:**

Our study helps to unravel the mechanism by which BCL-3 is linked to poor prognosis in colorectal cancer; we suggest that targeting BCL-3 activity represents an exciting therapeutic opportunity potentially increasing the sensitivity of tumour cells to conventional therapy.

**Significance of this study**

**What is already known on this subject?**

- The nuclear factor-κB (NF-κB) co-factor B-cell CLL/lymphoma 3 (BCL-3) was first identified in a t(14;19) translocation in a subgroup of B cell chronic lymphocytic leukaemias and has since been associated with the development of haematological neoplasias as well as solid tumour formation and growth.
- The NF-κB pathway plays an important role in the generation and maintenance of malignancies through the stimulation of cell survival, tumour progression, cell proliferation, angiogenesis and metastasis.
- Deregulation of the PI3K/AKT pathway due to gene amplification, activating mutations or loss of the tumour suppressor phosphatase and tensin homolog (PTEN) has been observed in a plethora of human cancers such as colorectal, gastric, thyroid, lung, cervical and ovarian cancer.
- A strong correlation between nuclear BCL-3 expression and poor prognosis has been reported in colorectal cancer.

**What are the new findings?**

- Expression of BCL-3 increases the growth of colorectal cancer cells in vivo (dependent on interaction with NF-κB p50 or p52 homodimers).
- Suppression of BCL-3 expression increases apoptosis in colorectal tumour cells.
- BCL-3 expression increases colorectal tumour cell survival through activation of the AKT/PKB pathway.
- BCL-3/NF-κB complexes activate the AKT survival pathway in colorectal carcinoma cells through simultaneous activation of PI3K and mTOR signalling.
- The anti-inflammatory drug 5-aminosalicylic acid suppresses BCL-3 expression in colorectal tumour cells.

**How might it impact on clinical practice in the foreseeable future?**

- BCL-3 has been identified as a potent survival factor in colorectal carcinogenesis that links pro-inflammatory NF-κB signalling and the AKT survival pathway. Targeting BCL-3 expression represents a potential new therapeutic opportunity to not only inhibit key pathways involved in tumour progression but also to increase the sensitivity of cancer cells to conventional therapy.

## Introduction

Colorectal cancer remains the third most common cancer worldwide,^1^ accounting for about 694 000 deaths per year,^2^ highlighting the urgent need to understand the mechanisms that promote cancer development and discover new therapeutic targets for treatment and prevention. As well as key genetic and epigenetic events that drive colorectal tumorigenesis, recent studies highlight the importance of the tumour microenvironment not only in tumour development and progression (e.g. (compare to line 204), Kaidi *et al*;^3^ Petherick *et al*^4^) but also in the tumour response to chemopreventative/therapeutic agents, leading to significant interest in targeting the tumour microenvironment both for chemoprevention and therapy.^5^

One signalling pathway that is of particular importance in tumour promotion is the nuclear factor-κB (NF-κB) pathway,^6^ which plays a pivotal role in the generation and maintenance of malignancies^7^ ^8^ through stimulation of cell survival, tumour progression, cellular proliferation, angiogenesis and metastasis.^8–11^ Another important pathway deregulated in a plethora of human cancers including colorectal cancer is AKT signalling, either by gene amplification, mutation or loss of the tumour suppressor PTEN.^12^ Here we report a novel mechanism by which NF-κB impacts on the AKT pathway to promote tumour cell survival via regulation by the proto-oncogene B-cell CLL/lymphoma 3 (BCL-3).

BCL-3 was first identified in a t(14;19) translocation in a subgroup of B cell chronic lymphocytic leukaemias,^13^ which has since been reported in other cancers of the blood, bone marrow and lymph nodes, and associated with poor prognosis.^14^ Further, overexpression without translocation has been observed in multiple subtypes of non-Hodgkin's and Hodgkin's lymphomas.^15^ ^16^ Emerging evidence indicates BCL-3 is of importance in solid tumour formation and growth;^17^ both BCL-3 mRNA and protein are overexpressed in breast tumours and breast cancer cell lines.^18^ ^19^ Overexpression of BCL-3 has also been reported in nasopharyngeal carcinoma,^20^ endometrial cancer^21^ and colorectal cancer.^22^

BCL-3 is an atypical member of the ankyrin repeat-containing IκB family of NF-κB inhibitors.^23–25^ NF-κB describes a family of transcription factor complexes formed by the NF-κB/rel gene family that in mammals is made up of five members, RelA (p65), RelB, c-Rel, NF-κB1 (p105/p50) and NF-κB2 (p100/p52), which share an N-terminal rel-homology domain that is required for dimerisation into homodimers and heterodimers, DNA binding, nuclear translocation and interaction with IκB. The Rel subfamily, RelA, RelB and c-Rel, contains a C-terminal transactivation domain (TAD), which is necessary for activation of transcription. NF-κB1 (p105/p50) and NF-κB2 (p100/p52) both lack this TAD and homodimeric complexes have been associated with transcriptional repression unless bound to a co-factor such as BCL-3 that contains a TAD^26^ ^27^ (reviewed in Perkins^28^). Unlike the usual mode of action of IκBs that repress activation of NF-κB signalling by sequestering NF-κB dimers within the cytoplasm, BCL-3 can localise to the nucleus where it preferentially binds NF-κB homodimeric complexes (p50:p50; p52:p52). Interestingly, BCL-3 has also been described as a transcriptional repressor, enhancing the binding of repressive p50 dimers to DNA.^29^ ^30^

A study by Puvvada *et al*^22^ showed an association between BCL-3 expression and clinical outcome in colorectal cancer; a strong correlation between nuclear BCL-3 expression and poor prognosis was reported. More recently, a paper by Liu *et al*^31^ reports that BCL-3 plays a critical role in stabilising c-MYC protein via ERK activation; deregulated c-MYC being of particular importance in colorectal carcinogenesis.^32^ ^33^ However, despite these striking observations, there remain few studies addressing how the expression of BCL-3 might contribute to colorectal carcinogenesis. Here we establish that BCL-3 is a potent survival factor in colorectal carcinogenesis, particularly in the context of stresses related to the tumour microenvironment. We provide evidence that BCL-3/NF-κB complexes act as novel activators of the antiapoptotic AKT signalling pathway that has been implicated in the molecular pathogenesis of a variety of human malignancies, including colorectal carcinogenesis.^11^ As BCL-3 is known to be induced by inflammatory cytokines^34^ ^35^ targeting BCL-3 expression (e.g. using non-steroidal anti-inflammatory drugs (NSAIDs)) would not only inhibit a key pathway involved in colorectal tumour progression but could also prevent some of the earliest events in inflamed tissue that drive colorectal tumorigenesis.

## Materials and methods

### Cell line and cell culture conditions

The human colorectal carcinoma-derived cell lines HCT116, HCA7 and SW480 were obtained from the American Type Culture Collection (ATCC, Maryland, USA) and maintained as previously described.^36^ HCT116 cells were cultured in McCoy's 5A Medium (Invitrogen, UK), whereas SW480 and HCA7 cells were grown in Dulbecco's Modified Eagle's Medium (DMEM; Source Bioscience, UK), both supplemented with 10% fetal bovine serum (FBS), 2 mM glutamine (Sigma, UK), 100 U/mL penicillin and 100 μg/mL streptomycin (Invitrogen). The non-tumorigenic adenoma-derived clonogenic S/RG/C2 cell line (referred to as RG/C2^37^ ^38^) was maintained in DMEM supplemented with 20% FBS, 2 mM L-glutamine (Sigma), 100 U/mL penicillin and 100 μg/mL streptomycin (Invitrogen) as well as 0.2 U/mL insulin (Actrapid Novo Nordisk, Denmark) and 1 μg/mL hydrocortisone (Sigma).

### Apoptosis assay

The induction of apoptosis was measured by determining the proportion of total cells that had detached from the epithelial monolayer and that were shed into the medium. Biochemical confirmation of apoptosis was obtained by the presence of cleaved poly (ADP-ribose) polymerase (PARP) in the shed cell population by western blotting and characteristic apoptotic morphology as demonstrated by acridine orange staining (described in detail previously^39^). Apoptosis was further validated using the pan caspase inhibitor QVD (5 μM) (551476; Calbiochem, EMD Millipore, UK), a broad-spectrum caspase inhibitor.

### RNAi

Cells were reverse transfected using Lipofectamine 2000 (Invitrogen) with small interfering RNAs (siRNAs; 50 nM) from Dharmacon (Thermo Fisher, UK) targeting BCL-3, NF-κB1 or NF-κB2. A single sequence was chosen to silence BCL-3 (specificity confirmed using a second sequence, data not shown), whereas NF-κB1 and NF-κB2 expression was silenced with pooled siRNAs; a single-sequence or pooled negative siRNA was used respectively.

### Plasmid DNA transfection

The wildtype (wt) BCL-3 and mutant BCL-3 expression plasmids^40^ ^41^ were a kind gift from Alain Chariot (University of Liège, Belgium). The mutant BCL-3 protein cannot bind NF-κB p50 and p52 homodimers due to three mutations in the ankyrin-repeat domain (BCL-3 ANK M123). The BCL-3 expression plasmids were tagged with a FLAG-tag and provided in pcDNA3 vectors. Cells were transiently transfected using Lipofectamine 2000 (Invitrogen) using empty pcDNA3 plasmid as a control. Stable BCL-3 overexpressing SW480 cells and their control were established using the same plasmids; resistant colonies were selected with 500 μg/mL neomycin and pooled.

We confirmed loss of NF-κB binding by the ANK M123 mutant in colorectal cancer cells using confocal microscopy. The wtBCL-3 increased nuclear localisation of both NF-κB1 and NF-κB2, whereas the mutant BCL-3 protein failed to do so (data not shown). Further, because the ANK M123 mutation affects BCL-3 protein stability, the amount of wt and mutant BCL-3 expression plasmid added was adjusted and the inability of the mutant BCL-3 expression construct to stimulate tumour necrosis factor-α-induced NF-κB activity was also verified (see online supplementary figure S1).

### Transient co-transfection with siRNA and plasmid DNA

Cells were reverse transfected with BCL-3 or negative siRNA (50 nM, Dharmacon, Thermo Fisher) as previously described. Following a recovery period of 24 h, cells were transfected with pcDNA3 expression plasmid encoding myristoylated constitutively active AKT (myr-AKT) using Lipofectamine 2000 (Invitrogen). An empty pcDNA3 plasmid was used as a negative control.

### Immunoblotting

Whole-cell lysates were prepared and subjected to immunoblotting as previously described^38^ using antibodies against BCL-3 (sc-185), NF-κB1 (p105/p50) (sc-8414; Santa Cruz Biotechnology, California, USA), p-AKT S473 (4058) and T308 (4056), total AKT (9272), p-FOXO1/3a (9464), p-GSK-3α/β (9331; Cell Signalling Technology, Massachusetts, USA), NF-κB2 (p100/p52) (05-361; EMD Millipore), HIF-1α (610958; BD Biosciences, UK). Equal loading was confirmed with α-tubulin (T9026; Sigma). Blots were quantified using ImageJ software (RSB, NIH, Maryland, USA).

### Immunofluorescence

Proteins were visualised as previously described^42^ using antibodies against p-AKT S473 (4058; Cell Signalling Technology), FLAG (F1804, Sigma). Cells were imaged at 100× magnification using a Leica AOBS SP2 confocal imaging system attached to a Leica DMI 6000 inverted microscope with Leica LCSLite software (Leica, Germany).

### Transwell assay

In brief, SW480 cells were seeded in a transwell insert with a permeable membrane on the bottom and grown for 48 h prior to transfection with wtBCL-3 or empty control plasmid DNA as described. The insert was rinsed in standard growth medium to avoid carryover of transfection reagent and placed onto untransfected SW480 cells growing in a six-well plate. Transfected and untransfected cells shared the same growth medium while being physically separated by the permeable membrane for 72 h prior to lysis.

### Immunohistochemistry

Immunohistochemistry (IHC) was carried out as previously described;^36^ formalin-fixed, paraffin-embedded tissue was obtained from the Bristol Royal Infirmary, Bristol, UK, with local Ethics Committee approval. BCL-3 was detected using the mouse monoclonal antibody (1E8, 1:50 dilution; Abcam, UK), NF-κB1 p105/p50 using (ab119826, 1:50 dilution; Abcam).

### Treatment of cells

The PI3K signalling pathway was inhibited with 20 nM Wortmannin (W1628; Sigma) while the mTOR signalling pathway was inhibited using 100 nM PP242 (P0037; Sigma). DMSO was added as a vehicle control at respective concentrations.

Cells were treated with 20 mM 5-aminosalicylic acid (5-ASA) (A3537; Sigma), a relevant luminal concentration in the colon.^43–45^

### Cell yield and cell death determination

Cell counts were made in triplicate, the attached cells were counted as a determination of cell yield and the detached cells as a measurement of cell death as previously described in detail.^39^ ^46^ Adherent and floating cells were pooled, fixed in 70% ethanol and stained with 50 μg/mL propidium iodide (P4170; Sigma), in the presence of RNase A (R6513; Sigma). Flow cytometry was performed on a LSR-II flow cytometer and data collected using FACDiva software (BD Biosciences). The population of cells exhibiting sub-G1 DNA was determined using ModFit LT software (Verity Software House, Maine, USA).

### Injection of nude mice

Six athymic nude mice (8–10 weeks) each were injected subcutaneously with 1×10^7^ SW480 cells stably expressing wtBCL-3, mutant BCL-3 or empty control plasmid DNA. Mice were kept under specific pathogen-free conditions, food and water ad lib. When the first tumour reached the maximum allowed diameter (16 mm), all mice were culled.

Xenotransplanted tumours were measured by calliper every 5 days for 30 days, and tumour volume was calculated according to (length×width×[length+width]/2). Mean values were calculated and plotted with analysis of variance.

### Statistical analysis

Statistical analysis was performed using Student's t test in Microsoft Office Excel 2007 unless otherwise stated and represented as *p<0.05; **p<0.01; ***p<0.001; NS=non-significant. Results are expressed as mean values with SD.

## Results

### BCL-3 is expressed in colorectal tumour cells in vivo and in vitro

**Figure 1:**
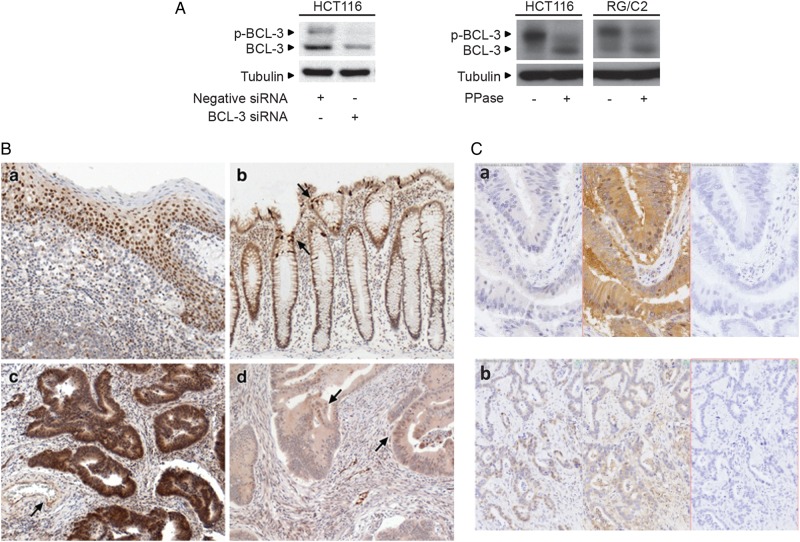

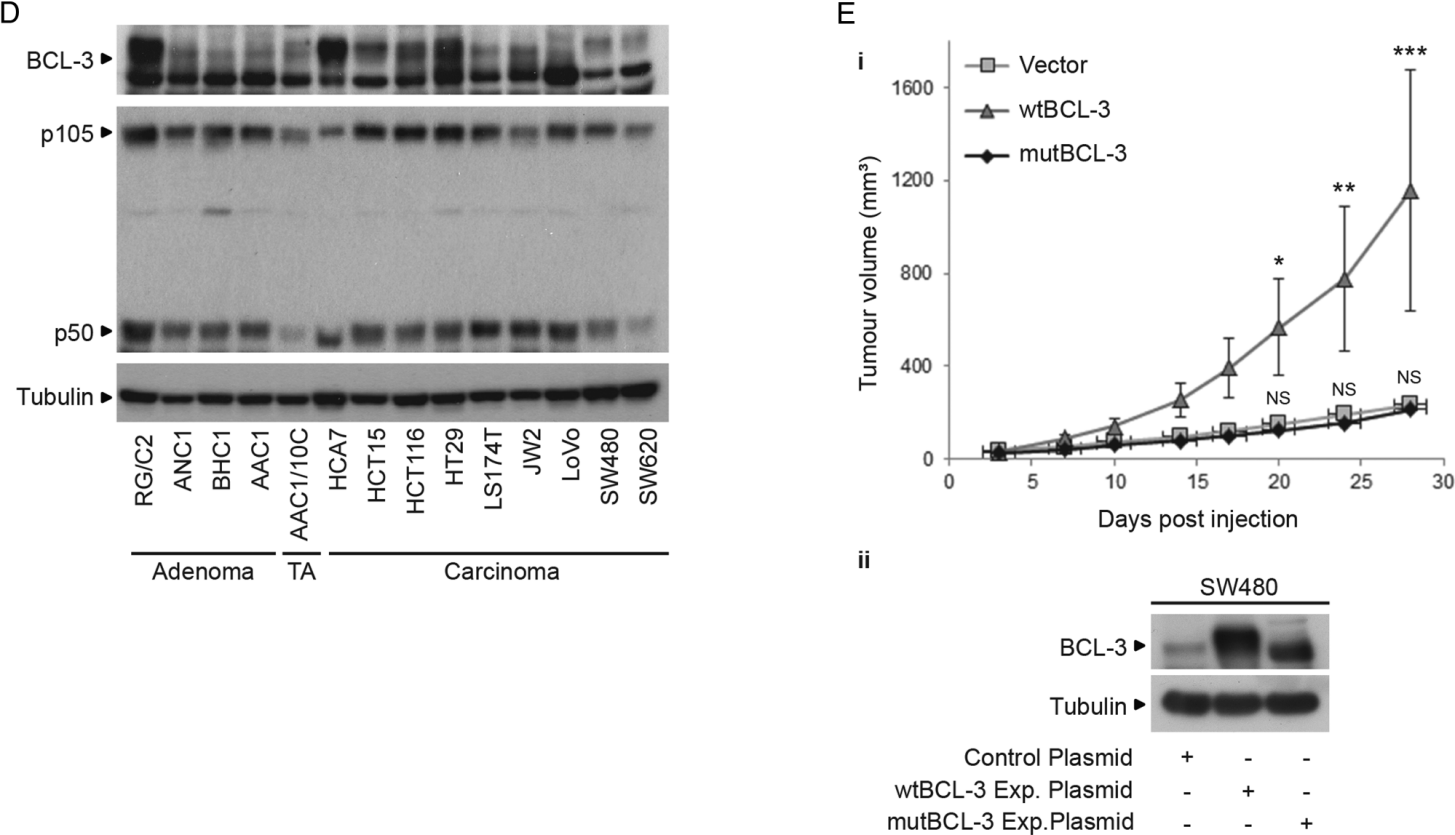
B-cell CLL/lymphoma 3 (BCL-3) is expressed in colorectal tumour tissue and cell lines and BCL-3 promotes the growth of colorectal tumour cells in vivo*.* (A) Western blot showing validation of BCL-3 antibody; the BCL-3 antibody used in this study detects two distinct bands that represent the BCL-3 protein in its phosphorylated (p-BCL-3) and unphosphorylated form (as shown in cells treated with lambda-phosphatase, 400 units for 2 h). (B) Immunohistochemistry (IHC) staining: (a) tonsil positive control for BCL-3 immunoreactivity yields strong nuclear staining of all but superficial keratinocytes, along with a scattered subset of inflammatory cells. (b) Normal colon showing moderate BCL-3 immunoreactivity in epithelial cell cytoplasm, with occasional strong positive cells (arrows). (c) Area of carcinoma showing strong cytoplasmic and nuclear BCL-3. The endothelium of the artery shows nuclear and cytoplasmic immunoreactivity (arrow). (d) In this tumour sample, the bulk of the tumour glands show weak or absent BCL-3 immunoreactivity, although foci of nuclear positivity are present (arrows). Original objective magnification; a×20; b–d×10. (C) IHC staining: (a) area of carcinoma with no staining for BCL-3 (left panel), strong cytoplasmic immunoreactivity for nuclear factor (NF)-κB1 (centre) and no staining in the absence of either primary antibody (right). (b) Area of carcinoma with nuclear BCL-3 staining (left panel), NF-κB1 staining (centre) and no staining in the absence of either primary antibody (right): no overall correlation between BCL-3 and NF-κB1 staining was detected. (D) Western analysis to determine BCL-3 and NF-κB1 protein expression in a panel of colorectal adenoma-derived and carcinoma-derived cell lines. TA, transformed adenoma. (E) Graph representing SW480 tumour growth in athymic nude mice: groups of six nude mice were injected with SW480 cells stably expressing either wildtype (wt)BCL-3, mutant (mut)BCL-3 expression or vector control plasmids (ii shown by western blotting) and tumour size was measured over 30 days. Mean values were plotted with SDs. Analysis of variance ***p<0.001, **p<0.01, *p<0.05, NS, non-significant. Note: the phosphorylation status and therefore the stability of the mutBCL-3 protein is affected by the ANK M123 mutation (Alain Chariot, personal communication, 2011); to compensate, the amount of expression plasmid was adjusted accordingly (refer to online supplementary figure S1).

We first demonstrated the specificity of the BCL-3 antibody using BCL-3 siRNA (figure 1A); two bands representing the unphosphorylated and phosphorylated forms of the protein were detected (phosphorylated BCL-3 was verified by treating the cell lysate with lambda-phosphatase, changing the two bands to the single unphosphorylated lower band). IHC was carried out on 47 tumour samples and expression compared with regions of normal tissue from the resection margins (examples are shown in figure 1B, tonsil tissue was included as a positive control for BCL-3 staining, panel a). Normal regions of colorectal mucosa showed weak/moderate cytoplasmic staining for BCL-3 and few nuclei exhibiting immunoreactivity, with the exception of scattered crypt cells that consistently showed strong positivity for BCL-3 (shown in figure 1B, panel b). Notably, the intensity of BCL-3 expression in colorectal carcinoma tissue was found to be more heterogeneous than in the normal tissue (with regions of high and low expression within the same tumour) although BCL-3 expression was generally increased (consistent with previous reports^22^ ^31^), with cytoplasmic staining of BCL-3 a consistent feature in 31 of the 47 colorectal cancer specimens. Interestingly, of these, 17 cancers also showed moderate/strong nuclear staining. An example of a very high staining area of carcinoma is shown in figure 1B (panel c), whereas figure 1B (panel d) shows an example of weak or absent BCL-3 expression, with foci of nuclear BCL-3 positivity. As BCL-3 is known to bind NF-κB p50 homodimers increasing its nuclear localisation,^26^ ^27^ we investigated whether there was a correlation between levels of BCL-3 and NF-κB1 expression in the tumours. Previous IHC studies have shown NF-κB1 expression in normal colonic tissue^47^ as well as more frequent p50 nuclear staining in tumour tissue versus normal mucosa.^22^ Six blocks of tissue from our series that had been scored strong (n=3) or weak (n=3) for BCL-3 immunoreactivity were stained again for BCL-3 and NF-κB1 (examples shown in figure 1C). Hamamatsu scans of BCL-3, NF-κB1 and control stained sections were compared side-by-side to seek any association between staining patterns. No correlation between expression of BCL-3 and NF-κB1 was found; due to a lack of suitable antibodies, any correlation with NF-κB2 could not be investigated. These results show that BCL-3 is expressed in normal epithelium and colorectal cancer, although there was no clear correlation with NF-κB1 expression as previously reported.^22^

BCL-3 expression was also assessed in a panel of colorectal adenoma-derived and carcinoma-derived cell lines by western blotting. BCL-3 was detected in all 14 cell lines investigated (figure 1D). The presence of NF-κB1 (p105/p50) (figure 1D) and NF-κB2 (p100/p52) (see online supplementary figure S1C) was also established in the cell lines. These findings show that both colorectal adenoma-derived and carcinoma-derived epithelial cells express BCL-3, NF-κB1 and NF-κB2. Given the importance of NF-κB in tumorigenesis and, taken together with the in vivo data showing increased expression of BCL-3 in a subset of tumours (previously associated with poor prognosis^22^), these results support a possible function for BCL-3:NF-κB homodimeric complexes in colorectal tumorigenesis.

### BCL-3 promotes the growth of colorectal tumour cells in vivo

As BCL-3 is an established NF-κB homodimer binding protein and NF-κB1 (p105/p50) is expressed in colorectal tissue,^22^ we studied the involvement of BCL-3:NF-κB complexes in colorectal cancer cell growth in vivo. Groups of six athymic nude mice were injected with pooled colonies of SW480 cells expressing either wtBCL-3 or a mutant BCL-3 protein unable to bind either NF-κB p50 or p52 homodimers^40^ ^41^ (BCL-3 ANK M123, a kind gift from Alain Chariot, University of Liège, Belgium; see online supplementary figure S1A). The effects of BCL-3 expression on the tumorigenicity of the cells in vivo are shown in figure 1E. Importantly, expression of wtBCL-3 significantly increased tumour size in athymic nude mice from 20 days post inoculation (figure 1E, in agreement with the findings of Liu *et al*,^31^ who report that suppression of BCL-3 in the CT26 CRC cell line inhibited tumour growth). Importantly, no difference in tumour size could be detected in athymic mice injected with vector control or mutant BCL-3-transfected SW480 cells, suggesting BCL-3:NF-κB homodimer binding is important for BCL-3 function.

### BCL-3 promotes tumour cell survival

**Figure 2:**
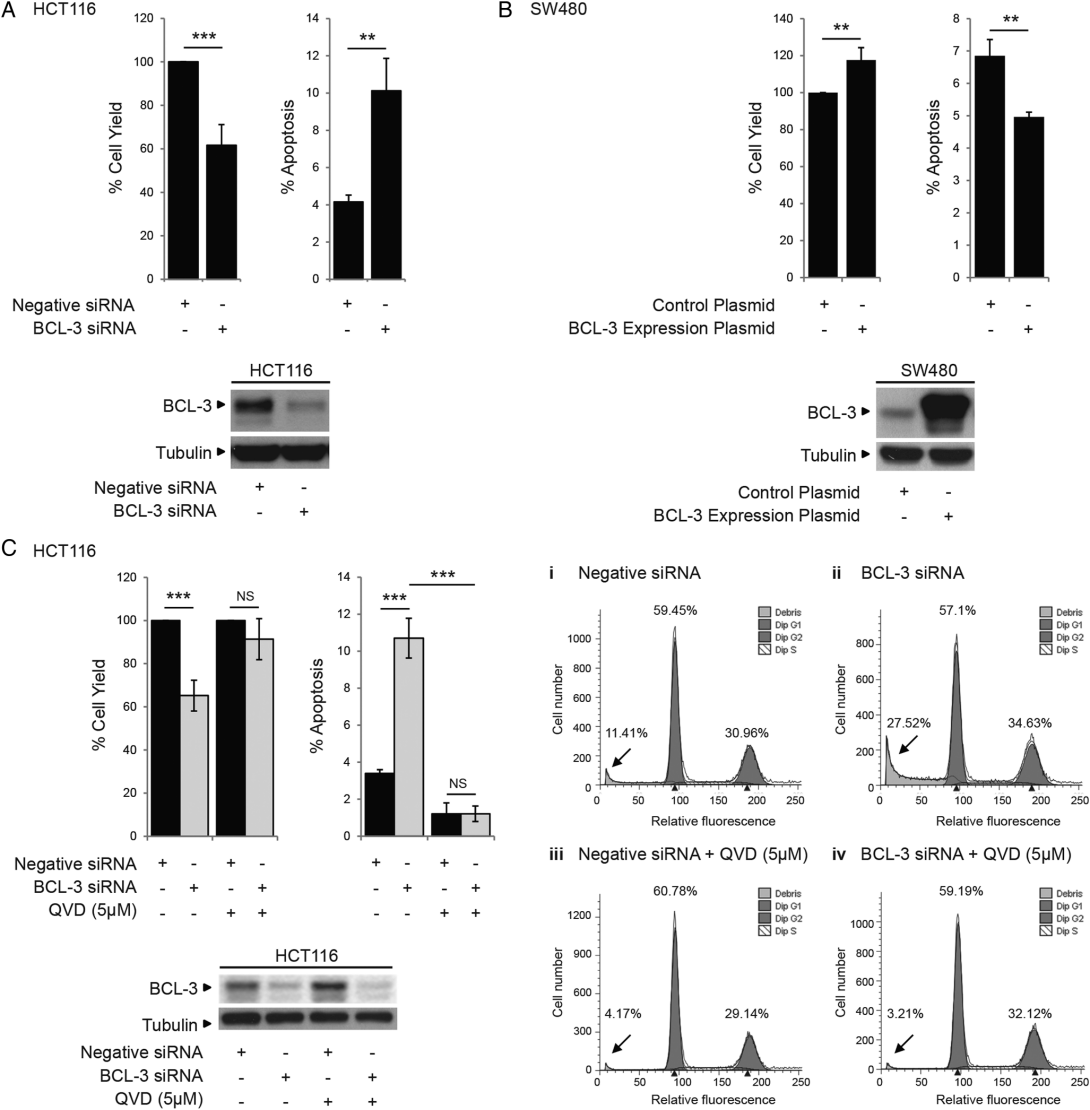

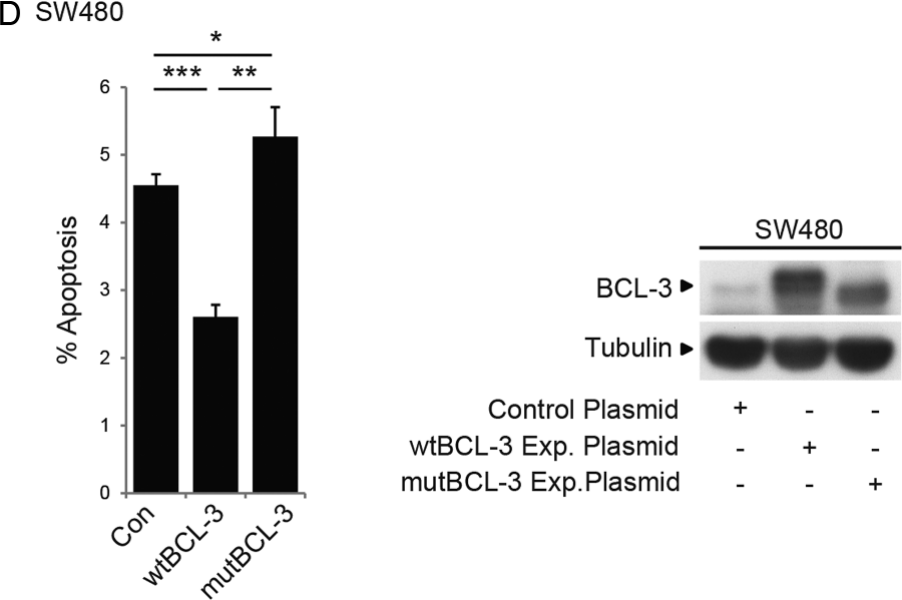
B-cell CLL/lymphoma 3 (BCL-3) expression inhibits apoptosis in colorectal cancer cells. (A) Suppression of BCL-3 expression significantly decreased the total cell yield and increased cell death compared with negative small interfering (si)RNA-transfected HCT116 cells 72 h post transfection. Suppression of BCL-3 protein expression was confirmed by western analysis. (B) BCL-3 overexpression significantly increased the total cell yield and decreased cell death compared with control plasmid-transfected SW480 cells 72 h post transfection. Overexpression of BCL-3 was confirmed by western analysis. (C) Treatment with 5 μM QVD (pan-caspase inhibitor) for 72 h reduced cell death significantly in control and BCL-3 knockdown HCT116 cells confirming induction of apoptosis following suppression of BCL-3 expression (***p<0.001, **p<0.01, NS, non-significant). Western analysis confirmed suppression of BCL-3 expression in QVD-treated HCT116 cells. Fluorescence-activated cell sorting analysis showed a significant increase in apoptosis (determined as sub-G1 peak, arrows) from 11.4% to 27.5% following BCL-3 suppression. In cells treated with QVD, apoptosis is inhibited in both control and BCL-3 knockdown cells (arrows). Results are representative of a single experiment performed in duplicate, assessed 72 h post transfection. (D) The decrease in cell death on stable overexpression of wildtype (wt)BCL-3 was not detected when the ANK M123 mutant (mut)BCL-3 was stably overexpressed compared with control plasmid-transfected SW480 cells 72 h after seeding. Overexpression of BCL-3 and mutBCL-3 was confirmed by western analysis.

**Figure 3:**
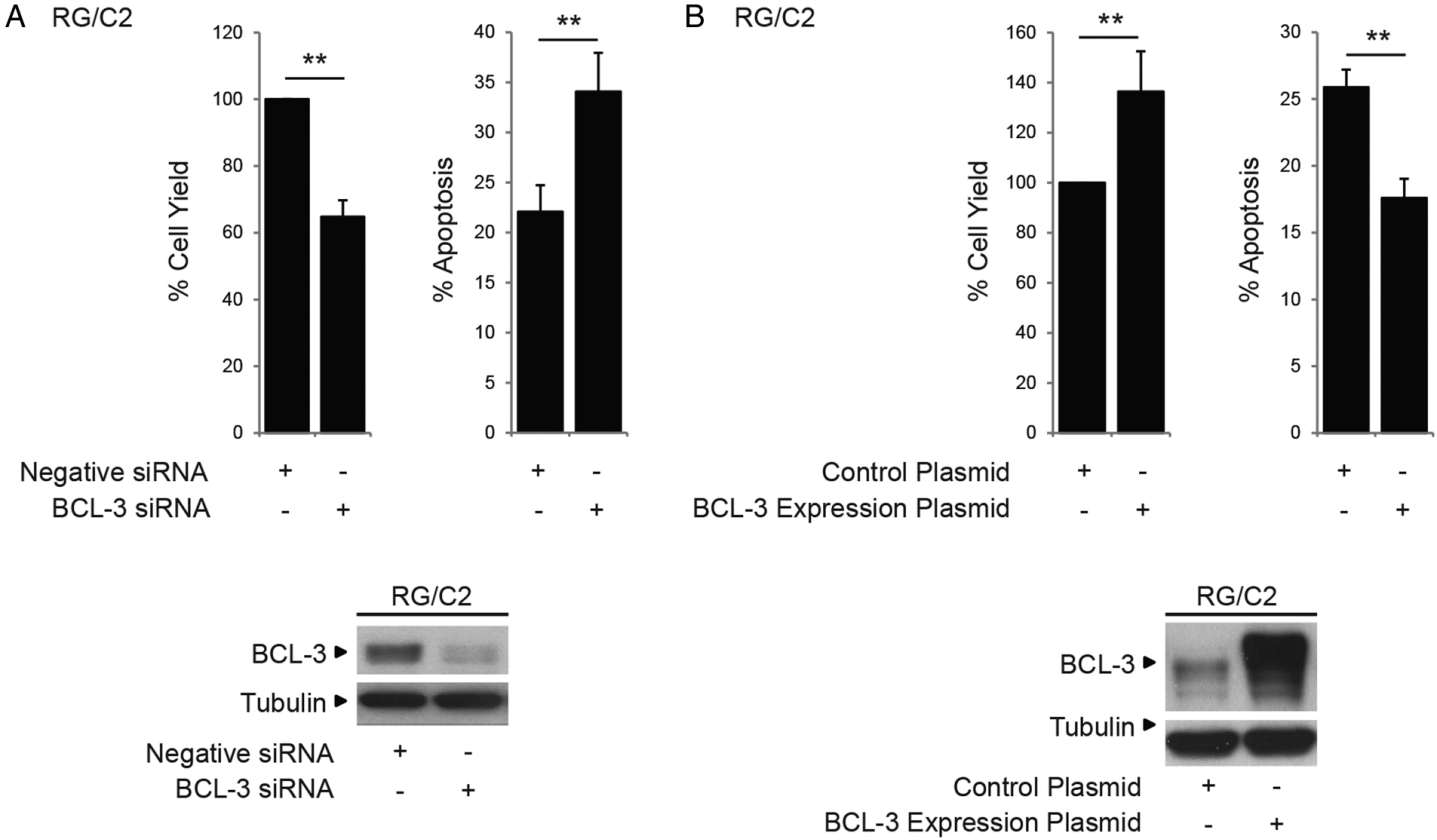
B-cell CLL/lymphoma 3 (BCL-3) expression promotes survival in colorectal adenoma cells. (A) RG/C2 adenoma cells were transfected with BCL-3 or negative small interfering (si)RNA and survival measured 72 h post transfection. Suppression of BCL-3 expression significantly decreased the total cell yield and increased cell death compared with negative siRNA-transfected RG/C2 cells. Suppression of BCL-3 protein expression was confirmed by western analysis. (B) RG/C2 cells were transfected with BCL-3 expression or control plasmid (1 μg) and survival measured 72 h post transfection. BCL-3 overexpression significantly increased the total cell yield and decreased apoptosis compared with control plasmid-transfected RG/C2 cells. Overexpression of BCL-3 was confirmed by western analysis.

To further examine the role of BCL-3 expression in promoting colorectal tumour cell growth, BCL-3 expression was suppressed using an siRNA approach in HCT116 cells (high endogenous BCL-3) and overexpressed in SW480 cells (low endogenous BCL-3) (figure 2). Suppression of BCL-3 led to a significant drop in total cell yield (40%) and increase in apoptosis (2.5-fold, figure 2A) further confirmed by complete inhibition using the pan-caspase inhibitor QVD and loss of the sub-G1 peak (figure 2C). In contrast, BCL-3 overexpression increased the cell yield and decreased cell death in SW480 cells (figure 2B). In addition, expression of the mutant BCL-3 (ANK M123) failed to suppress cell death, further suggesting BCL-3:NF-κB binding is important for BCL-3 function (figure 2D). It is important to note that similar results were observed in the non-malignant adenoma-derived RG/C2 cell line (figure 3). Taken together, the data demonstrates that BCL-3 expression increases colorectal tumour cell survival through suppressing apoptosis.

### BCL-3 inhibits apoptosis through activation of the AKT signalling pathway

**Figure 4:**
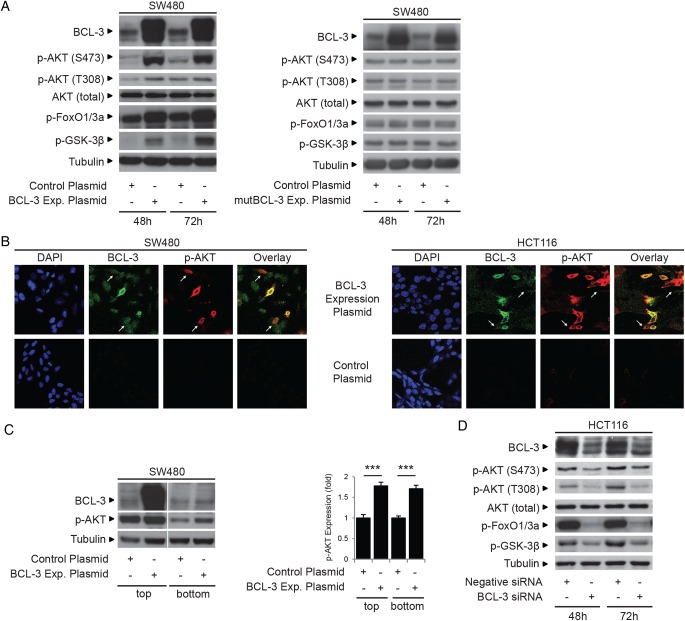

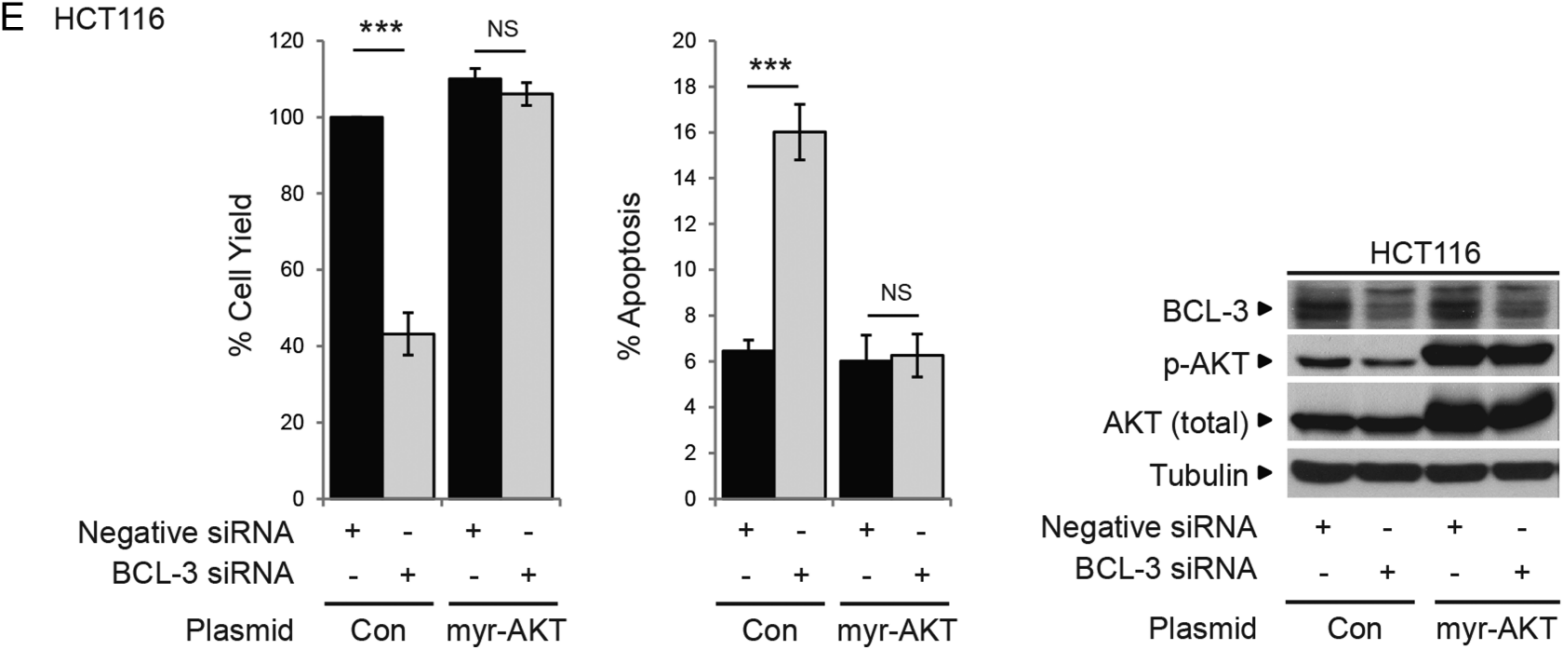
Phosphorylation of AKT and its downstream targets is increased in cells expressing high levels of B-cell CLL/lymphoma 3 (BCL-3): pro-survival effect of BCL-3 expression is mediated by AKT activation. (A) Western analysis of AKT, FoxO1/3a and GSK-3β phosphorylation following increased expression of wildtype (wt)BCL-3 or the ANK M123 mutant (mut)BCL-3. SW480 cells were transfected with wtBCL-3 or mutBCL-3 expression or control plasmids. Overexpression of wt and mutBCL-3 was confirmed (48–72 h post transfection). AKT phosphorylation at T308 and S473 was increased in cells overexpressing wtBCL-3 but not mutBCL-3 while levels of total AKT remained unchanged. Phosphorylation of AKT downstream targets FoxO1/3a and GSK-3β was also increased in the wtBCL-3 but not mutBCL-3-expressing cells. (B) Confocal microscopy of p-AKT (S473) (red) in SW480 and HCT116 cells transfected with FLAG-tagged BCL-3 (green) or control plasmid. Increased AKT phosphorylation was detected in HCT116 cells overexpressing BCL-3 as well as neighbouring cells with low BCL-3 (arrows). Cell nuclei were stained with 4′,6-diamidino-2-phenylindole (DAPI) (blue). (C) Cells grown in transwells (maintaining a physical separation between the two populations while allowing sharing of the growth medium) for 72 h prior to western analysis. Overexpression of BCL-3 was detected only in cells grown in the top chamber, leading to an increase in AKT phosphorylation (S473) compared with controls. AKT phosphorylation was also increased in untransfected SW480 cells sharing medium with cells overexpressing BCL-3. Samples run on the same western, quantification of AKT phosphorylation by densitometry, normalised to its control is shown. (D) Western analysis of AKT, FoxO1/3a and GSK-3β phosphorylation following suppression of BCL-3 expression. HCT116 cells were transfected with BCL-3 or negative small interfering (si)RNA. BCL-3 expression was suppressed throughout the time course (48–72 h post transfection). AKT phosphorylation at T308 and S473 was decreased in BCL-3 knockdown cells while levels of total AKT remained unchanged. Phosphorylation of AKT downstream targets FoxO1/3a and GSK-3β was also decreased. (E) Co-expression of constitutively active myristoylated AKT (myr-AKT) completely abrogates the induction of apoptosis following suppression of BCL-3 expression. HCT116 cells were transfected with BCL-3 or negative siRNA and subsequently myr-AKT or control plasmid. Suppression of BCL-3 expression decreased cell yield and increased apoptosis 72 h post transfection. HCT116 cells that lack BCL-3 but express myr-AKT show a similar percentage of apoptosis as control plasmid-transfected cells while no difference in the total cell yield could be detected, western analysis confirmed suppression of BCL-3. Strong AKT phosphorylation was detected in cells co-transfected with myr-AKT, ***p<0.001, NS, non-significant.

Given the importance of AKT as a potent survival factor in colorectal carcinogenesis,^12^ we addressed whether BCL-3 activated the AKT/PKB signalling pathway in the tumour cells. Interestingly, western analysis and confocal microscopy revealed that overexpression of BCL-3 (but not the mutant ANK M123 BCL-3 protein) promoted phosphorylation of AKT (S473 and T308) and known downstream targets including FoxO1/3a and GSK-3β (figure 4A). Of note, expression of BCL-3 was also able to increase p-AKT levels in some neighbouring cells (figure 4B, white arrows), supported by experiments where activation of AKT was detected in cells either grown in transwells with cells overexpressing BCL-3 (figure 4C) or exposed to conditioned media from BCL-3 overexpressing cells (data not shown). Further, suppression of BCL-3 expression in HCT116 cells resulted in reduced AKT phosphorylation and concurrent suppression of downstream target phosphorylation (figure 4D).

To show that the AKT pathway was responsible for the pro-survival effect of BCL-3, constitutively active myr-AKT was expressed in the HCT116 cells. Expression of myr-AKT blocked apoptosis induced by BCL-3 knockdown (figure 4E). Further, exposure of SW480 and HCT116 cells to low doses of an AKT1/2 inhibitor blocked the antiapoptotic function of BCL-3 (see online supplementary figure 2A, B). Similar results were obtained for the adenoma-derived cell line RG/C2 (see online supplementary figure 2C), confirming that BCL-3 acts as a potent survival factor through activation of the AKT/PKB signalling pathway in colorectal tumour cells.

**Figure 5:**
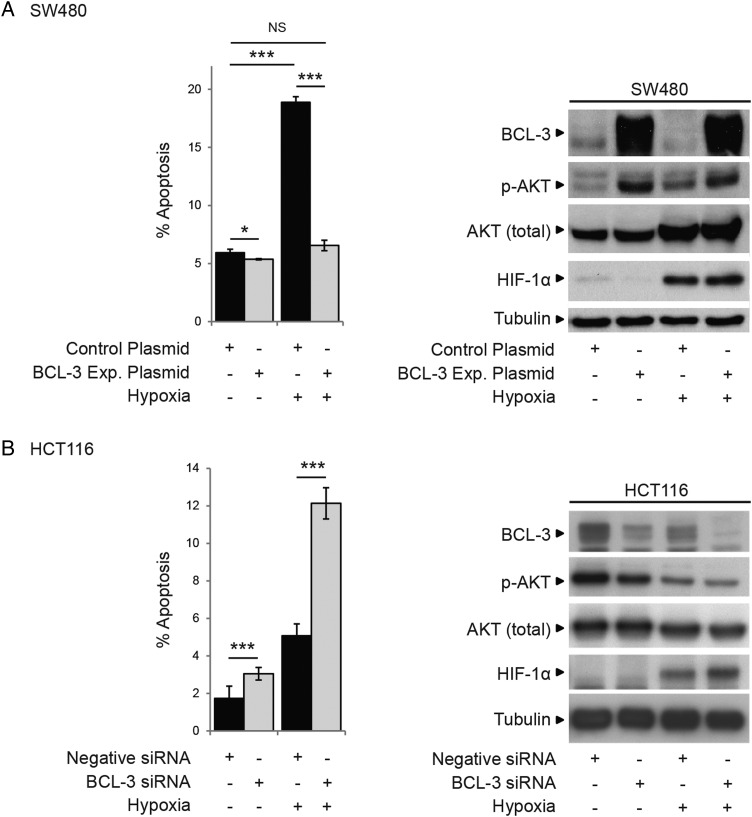
B-cell CLL/lymphoma 3 (BCL-3) expression promotes cell survival in hypoxic growth conditions. (A) SW480 cells transfected with BCL-3 expression or control plasmid were placed in 1% O_2_ for 48 h. Hypoxia significantly decreased cell yield and increased apoptosis in control plasmid-transfected cells. BCL-3 overexpression in 1% O_2_ decreased cell death to that observed for control plasmid-transfected cells in normoxia. Western analysis showed successful overexpression of BCL-3 in 1% O_2_. Expression of HIF-1α was detected in cells placed in 1% O_2_. Total AKT levels increased in 1% O_2_ (only seen in this cell line). AKT phosphorylation at S473 was increased in cells overexpressing BCL-3 in 1% O_2_ as well as normoxia. (B) HCT116 cells were transfected with BCL-3 or negative small interfering (si)RNA were placed in 1% O_2_ for 48 h. AKT phosphorylation at S473 was decreased in BCL-3 siRNA-transfected cells in hypoxia as well as normoxia, while levels of total AKT remained unchanged. Western analysis showed successful suppression of BCL-3 in 1% O_2_.

As we are interested in tumour cell survival in the context of the tumour microenvironment, it is of note that increased expression of BCL-3 in the SW480 cell line increased AKT phosphorylation (S473) and conferred resistance to apoptosis induced by hypoxia (figure 5A). Furthermore, suppression of BCL-3 expression in HCT116 cells decreased AKT phosphorylation in response to hypoxia and increased cell death (figure 5B). Hence, BCL-3 may be particularly important as a survival factor in colorectal carcinogenesis in the context of stresses related to the tumour microenvironment.

### BCL-3-dependent AKT activation is dependent upon BCL-3:NF-κB binding

**Figure 6:**
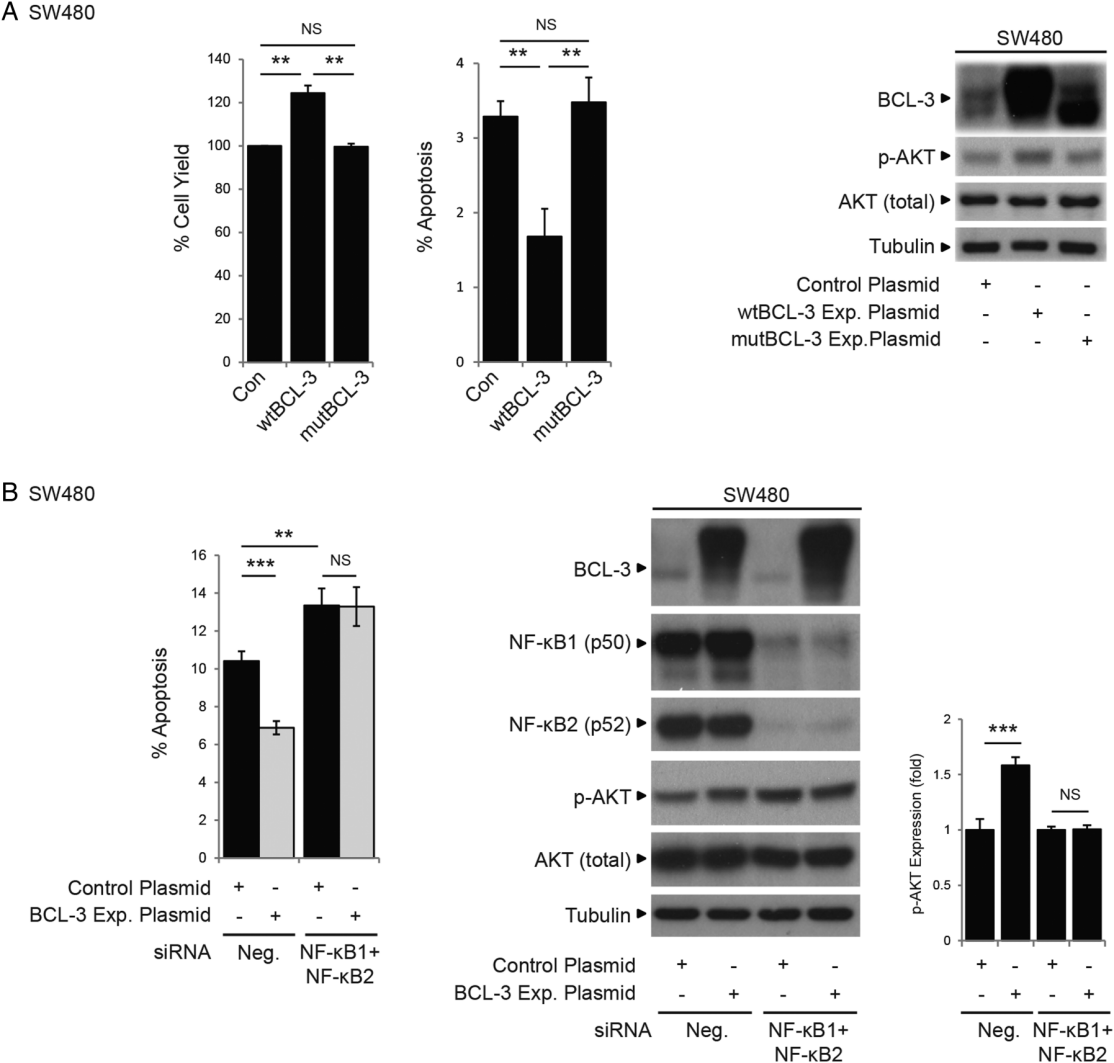
B-cell CLL/lymphoma 3 (BCL-3) increases survival of colorectal carcinoma cells that is dependent on nuclear factor (NF)-κB p50/p52 binding. (A) SW480 cells were stably transfected with wildtype (wt)BCL-3, mutant (mut)BCL-3 (NF-κB p50/p52 binding mutant) or control plasmid. The total cell yield was significantly increased and apoptosis decreased in cells overexpressing wtBCL-3 protein compared with mutBCL-3 or control plasmid-transfected cells. Western analysis showed AKT phosphorylation at S473 was only increased in cells overexpressing the wtBCL-3 protein. Note: the phosphorylation status and therefore the stability of the mutBCL-3 protein is affected by the ANK M123 mutation (Alain Chariot, personal communication, 2011); to compensate, the amount of expression plasmid was adjusted accordingly (refer to online supplementary figure S1). (B) SW480 cells were transfected with both NF-κB1 and NF-κB2, or negative small interfering (si)RNA and subsequently transfected with the BCL-3 expression or control plasmid. Apoptosis was decreased and AKT phosphorylation increased in cells transfected with the BCL-3 expression plasmid/negative siRNA compared with negative/negative controls. In contrast, no difference in the percentage of cell death and no increase in p-AKT levels could be detected between cells overexpressing BCL-3 and controls when both NF-κB1 and NF-κB2 expression were suppressed. Note: suppression of NF-κB1/NF-κB2 increased endogenous p-AKT that was not sufficient to overcome apoptosis induced by suppression of both NF-κBs. Importantly, no further increase in p-AKT was detected in BCL-3 overexpressing cells in the absence of NF-κB1 and NF-κB2. Quantification of AKT phosphorylation by densitometry normalised to its control is shown. Data was assessed 72 h post transfection. ***p<0.001, **p<0.01, NS, non-significant.

Although it is well established that both canonical and non-canonical NF-κB pathways have pivotal roles in carcinogenesis, the participation of the atypical NF-κB pathway (defined here as transcriptional regulation by NF-κB homodimeric complexes) is less well understood. As NF-κB-independent activity of BCL-3 has been reported,^17^ we investigated whether the pro-survival role of BCL-3 was dependent on NF-κB homodimeric binding. Using the mutant BCL-3 expression plasmid (ANK M123), we demonstrated that loss of NF-κB binding completely abrogated the antiapoptotic function of BCL-3 (as shown by the fact that mutant BCL-3 failed to increase cell survival and activate the AKT/PKB signalling pathway, figures 2 and 6A). Results obtained using the mutant BCL-3 protein were confirmed using an siRNA approach to simultaneously suppress NF-κB1 and NF-κB2 expression in SW480 cells overexpressing wtBCL-3; the antiapoptotic effect of BCL-3 was blocked and AKT activation absent (figure 6B). Taken together with the in vivo study (figure 1E), these results demonstrate that the pro-survival function of BCL-3 is dependent on NF-κB binding and suggest an important role for the BCL-3:NF-κB complexes in colorectal carcinogenesis.

### BCL-3-induced AKT activation is not regulated through altered PTEN expression

**Figure 7:**
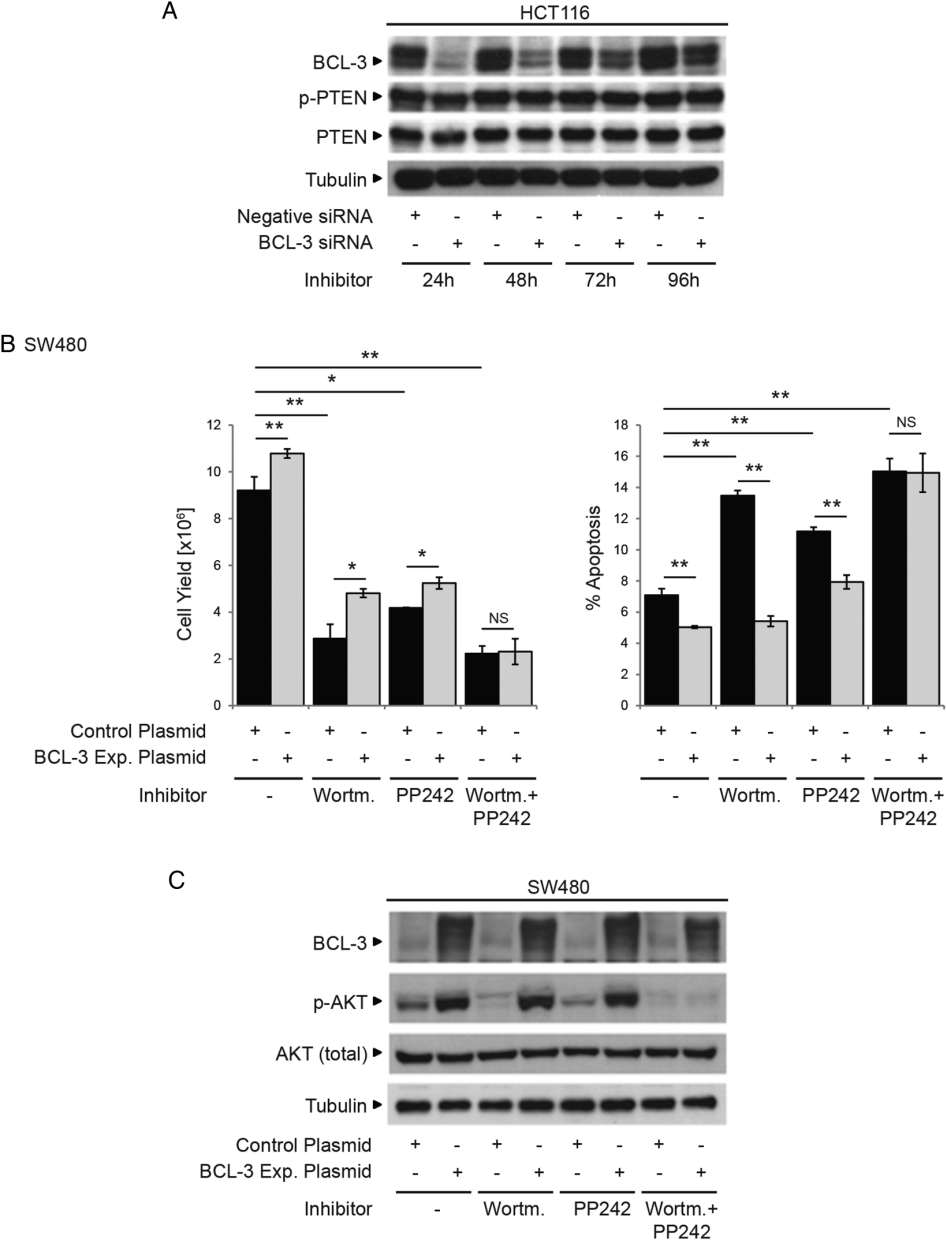
B-cell CLL/lymphoma 3 (BCL-3)-mediated survival is dependent on PI3K and mTOR activation. (A) HCT116 cells were transfected with BCL-3 or negative small interfering (si)RNA. Western analysis showing BCL-3 expression was suppressed throughout the time course (24–96 h post transfection). PTEN and phospho-PTEN expression remained unchanged throughout the time course of the experiment. (B) SW480 cells transfected with either BCL-3 expression or control plasmid were treated with 20 nM Wortmannin (PI3K inhibitor), 100 nM PP242 (mTOR inhibitor) or both inhibitors at the same time for 72 h. Overexpression of BCL-3 resulted in a significant increase in cell yield and decrease in apoptosis in untreated SW480 cells. Apoptosis was increased in Wortmannin (PI3K inhibitor) as well as PP242 (mTOR inhibitor) treated cells compared with untreated controls. No difference in apoptosis or cell yield could be detected comparing control and BCL-3 expression plasmid-transfected cells when PI3K and mTOR activation were simultaneously inhibited. Results are mean values from three independent experiments performed in duplicate normalised to controls, **p<0.01, *p<0.05, NS, non-significant. (C) Western analysis confirmed BCL-3 overexpression in treated and untreated cells. AKT phosphorylation (S473) is decreased in Wortmannin and PP242-treated control plasmid-transfected cells. p-AKT was increased in treated cells overexpressing BCL-3. In contrast, AKT phosphorylation was undetectable in cells simultaneously treated with Wortmannin and PP242. Data was assessed 72 h post transfection.

As BCL-3:NF-κB homodimeric complexes are regulators of gene transcription, the question remained whether AKT activation was regulated through suppression of PTEN (which is a negative regulator of AKT activity). Using BCL-3 suppression in HCT116 cells, we were able to show that BCL-3 expression does not regulate PTEN levels or activation (figure 7A). To further investigate the mechanism of BCL-3-dependent AKT activation, we studied the effect of BCL-3 expression on a number of pathways known to promote AKT phosphorylation (figure 7B, C). Results presented in figure 7 show that the combined treatment with the PI3K inhibitor Wortmannin and mTOR inhibitor PP242 prevented BCL-3-mediated AKT phosphorylation in SW480 cells while inhibition of either pathway alone did not block BCL-3-mediated AKT phosphorylation (figure 7B). These results show that BCL-3:NF-κB homodimeric complexes trigger the activation of PI3K and mTOR signalling to activate the AKT survival pathway in colorectal tumour-derived cells.

### Treatment with 5-ASA decreases BCL-3 expression

**Figure 8:**
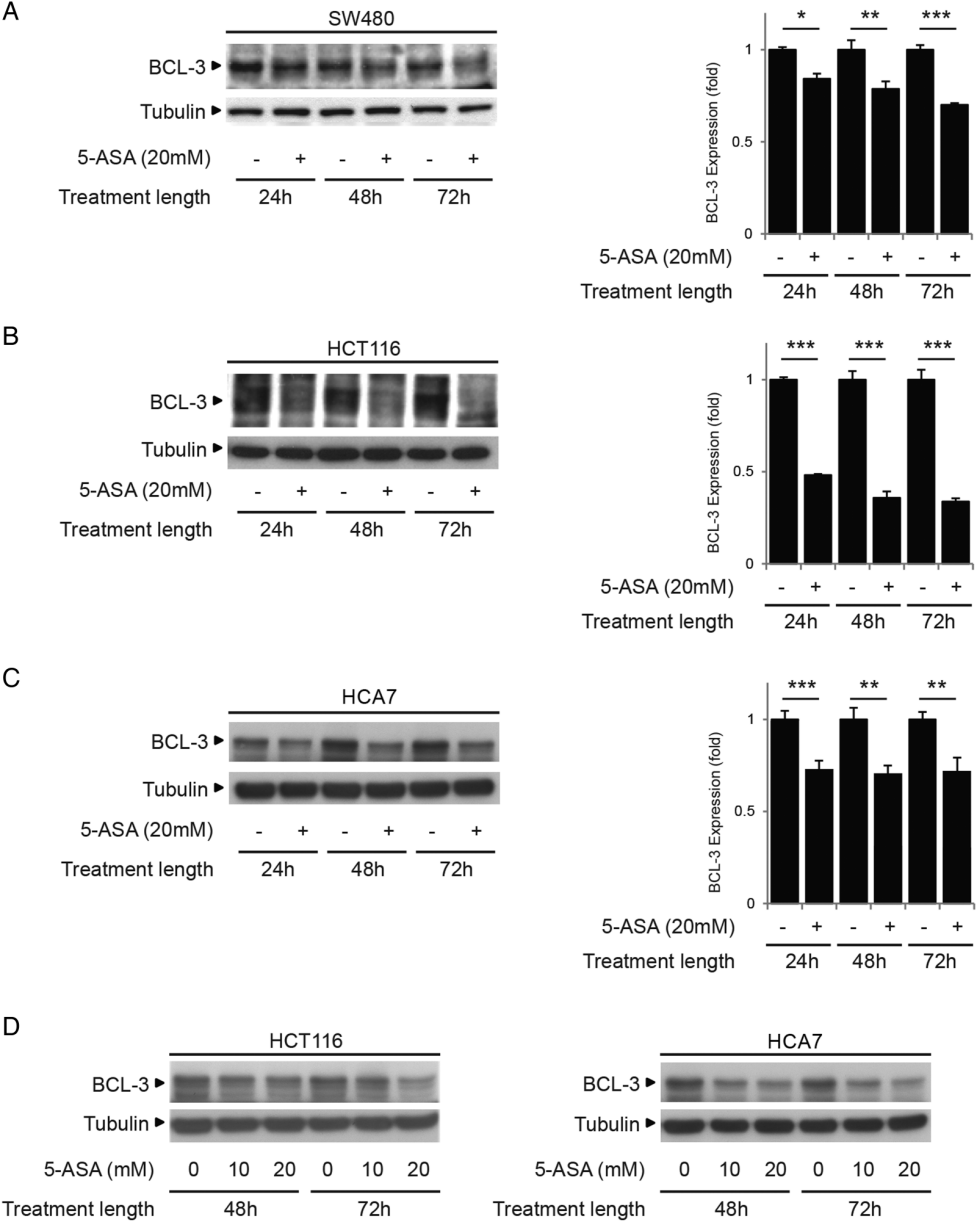
Treatment with 5-aminosalicylic acid (5-ASA) decreases B-cell CLL/lymphoma 3 (BCL-3) expression in colorectal cancer cells. Low BCL-3 expressing SW480 (A) and high BCL-3 expressing HCT116 (B) and HCA7 (C) cells were seeded for 48 h prior to treatment with 20 mM 5-ASA. Data was assessed at 24, 48 and 72 h post treatment. Western analysis showed expression of BCL-3 was decreased in all cells at 24, 48 and 72 h. Quantification of BCL-3 by densitometry normalised to its control is shown. (D) The high endogenous BCL-3 expressing cells (HCT116 and HCA7) were seeded for 48 h prior to treatment with 0, 10 or 20 mM 5-ASA. Western analysis shows BCL-3 expression was suppressed with 10 and 20 mM 5-ASA 48 h post treatment.

Epidemiological studies suggest that the aminosalicylate 5-ASA (Mesalazine) may reduce the risk of colorectal cancer in patients with UC.^48^ ^49^ It was therefore interesting to note that treatment with 5-ASA suppresses BCL-3 expression in a number of colorectal carcinoma cell lines (figure 8). BCL-3 expression was suppressed in SW480, HCT116 and HCA7 cells (figure 8A–C) with 20 mM 5-ASA treatment.^44^ ^45^ Further in the cells with higher endogenous BCL-3 expression (HCT116 and HCA7), suppression of BCL-3 was detected with doses as low as 10 mM 5-ASA (figure 8D).

## Discussion

We have shown for the first time that BCL-3 promotes the growth of colorectal cancer cells through activation of the AKT pathway, increasing tumour cell yield both in vitro and in vivo. Similar to the findings of Puvvada *et al*,^22^ and more recently Liu *et al*,^31^ we found BCL-3 expression was increased in colorectal cancer tissue, although it was often heterogeneous within the sample, with areas of strong staining adjacent to areas of moderate BCL-3 expression. We present evidence that this may be of particular importance in the context of surviving stresses such as hypoxia, encountered within the tumour microenvironment. As the pro-survival action of BCL-3 was also demonstrated in colorectal adenoma-derived cells, results suggest that targeting BCL-3 function may be effective both in the prevention and treatment of cancer.

The AKT/PKB pathway is an important signalling pathway involved in cell survival, inhibition of apoptosis, cell cycle progression and proliferation (reviewed in Zoncu *et al*^50^). In carcinogenesis, loss of PTEN is commonly observed in tumours of the colon.^51^ ^52^ Activated AKT phosphorylates numerous substrates such as GSK-3β and FoxO1/3a that are involved in the regulation of cell cycle progression, survival, protein translation and metabolism^53^ (reviewed in Carnero^54^). As AKT, through inhibiting GSK-3β, can also stabilise c-MYC, it was of interest that while this manuscript was in preparation, Liu *et al* published a study reporting that BCL-3 stabilises c-MYC in HCT116 colorectal cancer cells. Interestingly in contrast to the current study, they failed to link stabilisation with activation of AKT signalling, describing instead a phospho-ERK1/2-dependent mechanism (not detected in our cells, data not shown).^31^ As both our studies use HCT116 cells, it is difficult to resolve the differences in the findings, suggesting an element of context-dependent regulation. However, it is important to stress that in the current study AKT activation by BCL-3 was clearly detected in more than one carcinoma cell line (we have detected BCL-3-induced AKT activation in SW480, SW620, HCA7, HT29 and HCT116 cells) as well as RG/C2 adenoma-derived cells. Further, despite the fact that we could not show consistent regulation of c-MYC protein levels by BCL-3 in any of the cell lines (data not shown) the two studies describe potentially complementary mechanisms that clearly emphasise the importance of BCL-3 expression in colorectal tumour cell growth.

One advantage of BCL-3 as a possible therapeutic target is that it may impact on a number of different pro-survival pathways. It is of interest that activated AKT also phosphorylates IκB kinase (IKK), to directly activate NF-κB signalling.^55^ Indeed, crosstalk between AKT and NF-κB signalling has already been shown to promote cell survival.^55^ ^56^ Here we present a novel mechanism by which AKT activity can be increased in colorectal epithelial cells; our results suggest that potentiation of AKT signalling (observed in both adenoma-derived RG/C2 and five carcinoma-derived cell lines) is central to the pro-tumorigenic role of BCL-3. Interestingly, although the mechanism by which the BCL-3 complexes increase AKT signalling remains to be elucidated, data from the immunofluorescence study and co-culture experiments (figure 4) does suggest that increased expression of BCL-3 in one cell can effect phospho-AKT levels in neighbouring cells, leading to the hypothesis that the induction of BCL-3 would contribute to a pro-tumorigenic microenvironment. Combined with the observation that nuclear BCL-3 is associated with poor prognosis in patients with colorectal cancer,^22^ the fact that BCL-3 expression increased activation of the pro-survival AKT pathway and increased the growth of colorectal tumour cells in mice (xenografts) emphasises the potential importance of BCL-3 in colorectal carcinogenesis.

Finally, we make the interesting observation that the common NSAID 5-ASA can suppress BCL-3 expression in the tumour cells. As we have shown that BCL-3 acts as a survival factor in the adenoma as well as carcinoma-derived cells, it is interesting to speculate that the ability to repress BCL-3 may contribute to the effectiveness of these NSAIDs in preventing tumorigenesis.

In summary, we have shown that BCL-3 is a potent survival factor in colorectal adenoma-derived and carcinoma-derived cells and propose that the targeting of BCL-3 expression represents a potentially exciting therapeutic opportunity, inhibiting some of the key pathways involved in colorectal tumour progression.

## Supplementary Material

Web supplement

Web figures
