# Supplementary material for: BCL-3 expression promotes colorectal tumorigenesis through activation of AKT signalling
Source: Gut. 2015 Jun 1;65(7):1151–64. doi: 10.1136/gutjnl-2014-308270 (PMC4941180; doi:10.1136/gutjnl-2014-308270)
Supplement: Web supplement [file gutjnl-2014-308270-s1.pdf]

Supplementary Materials & Methods

NF-κB reporter assay

Cells were transiently transfected with wtBCL-3, mutBCL-3 [1, 2] or empty pcDNA plasmids as well as either the NF-κB reporter plasmid pNF-κB-TA-luc or the control plasmid pTA-luc (Clontech, Oxford, UK) and pRL-SV40 renilla plasmid (Promega, Southampton, UK) using Lipofectamine 2000 (Invitrogen, Paisley, UK) according to the manufacturer's protocol. Following lysis, luciferase activities were measured using the Dual-Luciferase Reporter Assay System (Promega, Southampton, UK) according to the manufacturer's instructions.

Treatment of cells

The AKT signalling pathway was inhibited using 5 nM AKT1/2 kinase inhibitor (A6730; Sigma, Poole, UK).

References:

1. Keutgens A, Shostak K, Close P, et al. The repressing function of the oncoprotein BCL-3 requires CtBP, while its polyubiquitination and degradation involve the E3 ligase TBLR1. Mol Cell Biol. 2010;30(16):4006-21.
2. Viatour P, Dejardin E, Warnier M, et al. GSK3-mediated BCL-3 phosphorylation modulates its degradation and its oncogenicity. Mol Cell. 2004;16(1):35-45.
