# Supplementary material for: BCL-3 expression promotes colorectal tumorigenesis through activation of AKT signalling
Source: Gut. 2015 Jun 1;65(7):1151–64. doi: 10.1136/gutjnl-2014-308270 (PMC4941180; doi:10.1136/gutjnl-2014-308270)
Supplement: Web figures [file gutjnl-2014-308270-s2.pdf]

Supplementary Figure 1

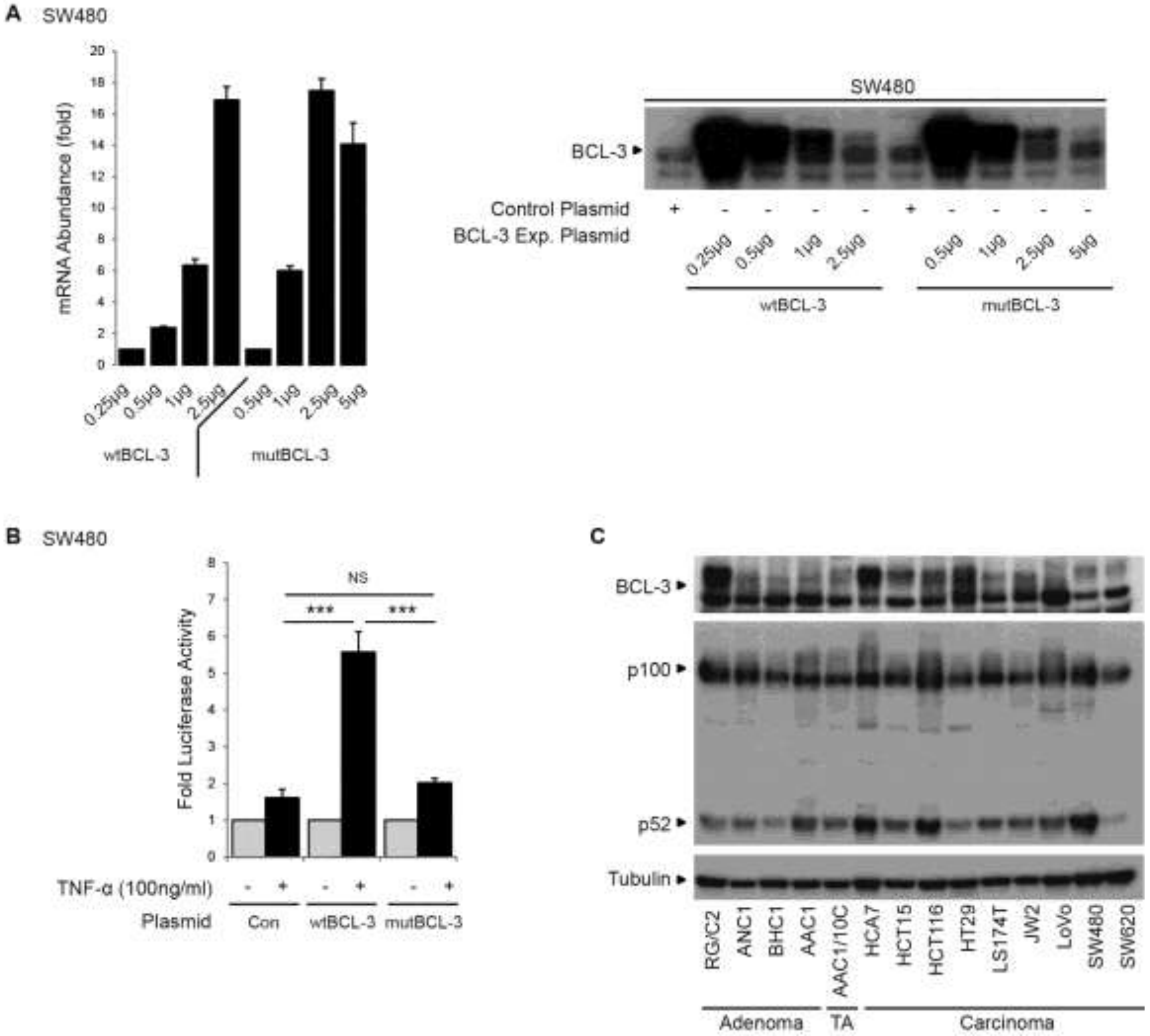

Supplementary Figure 1 | Comparable levels of wtBCL-3 and mutBCL-3 mRNA and protein are expressed in SW480 cells, and TNF-α induced NF-κB transcriptional activity is dependent on BCL-3:NF-κB binding.

(**A**) SW480 cells were transfected with different concentrations of wtBCL-3 and mutBCL-3 expression plasmid. Q-RT-PCR showed comparable levels of mRNA were present in SW480 cells transfected with 0.5-2.5µg wtBCL-3 or mutBCL-3 expression plasmid at 72h post transfection. Q-RT-PCR results were normalized to the lowest concentration examined. Equal levels of both wtBCL-3 and mutBCL-3 protein were expressed at 24h post transfection for 0.5 and 1µg plasmid. **(B)** Using an NF-κB reporter assay, overexpression of wtBCL-3 was shown to increase TNF-α-induced NF-κB transcriptional activity. In contrast, no increase in cytokine induced NF-κB transcriptional activity could be observed in cells expressing the mutBCL-3 protein compared to controls. Results are mean values from three independent experiments performed in triplicate normalized to controls, *** $P<0.001$, NS = non-significant. **(C)** Western analysis to determine BCL-3, and NF-κB2 protein expression in a panel of colorectal adenoma- and carcinoma-derived cell lines. TA refers to transformed adenoma.

Supplementary Figure 2

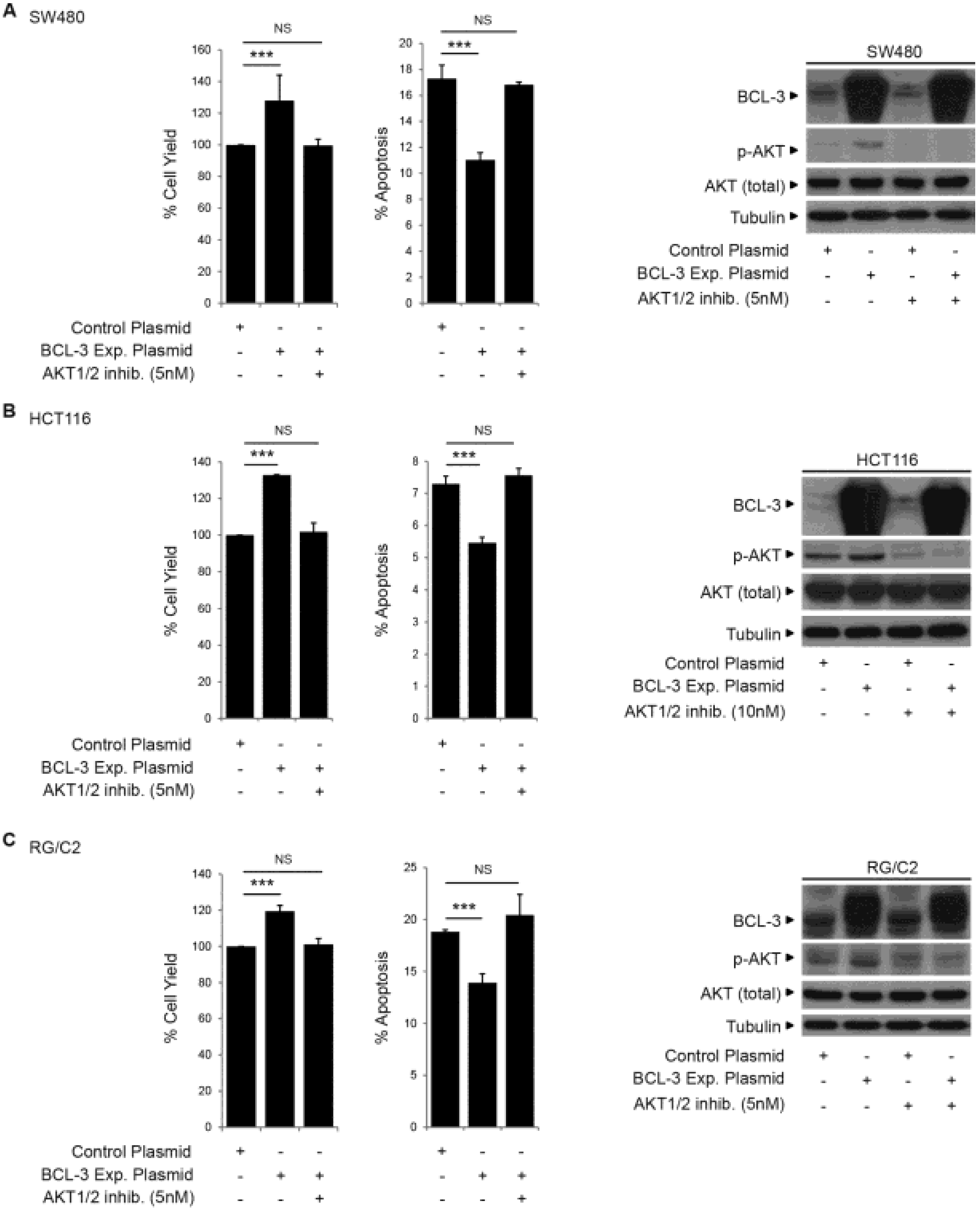

Supplementary Figure 2 | Pro-survival effect of increased BCL-3 expression is blocked by inhibition of AKT signalling.

(A)SW480, (B) HCT116 and (C) RG/C2 cells were seeded for 48h and transfected with BCL-3 expression or control plasmids (1μg). Cells were treated with 5nM AKT1/2 inhibitor for 96h post transfection before data was assessed. BCL-3 overexpression increased cell yield and decreased apoptosis in untreated cells. This survival advantage was completely lost in cells treated with the AKT1/2 inhibitor, with BCL-3 transfected cells showing the same percentage of apoptosis as treated controls. Results are mean values from three independent experiments performed in triplicate normalized to controls with standard deviations (*** $P<0.001$, NS = non significant). Western analysis shows that BCL-3 is overexpressed in both untreated cells and cells treated with the AKT1/2 inhibitor. AKT phosphorylation is increased in untreated cells overexpressing BCL-3, but completely lost in cells treated with the AKT1/2 inhibitor confirming successful inhibition of AKT activation. Equal loading was confirmed by α-tubulin.
